# Religious Fragmentation, Social Identity and Conflict: Evidence from an Artefactual Field Experiment in India

**DOI:** 10.1371/journal.pone.0164708

**Published:** 2016-10-21

**Authors:** Surajeet Chakravarty, Miguel A. Fonseca, Sudeep Ghosh, Sugata Marjit

**Affiliations:** 1 Department of Economics, University of Exeter, Exeter, United Kingdom; 2 NIPE, Universidade do Minho, Braga, Portugal; 3 Department of Economics, Hong Kong Polytechnic University, Hong Kong, Hong Kong; 4 Department of Economics, Center for Studies in the Social Sciences, Kolkata, West Bengal, India; Middlesex University, UNITED KINGDOM

## Abstract

We examine the impact of religious identity and village-level religious fragmentation on behavior in Tullock contests. We report on a series of two-player Tullock contest experiments conducted on a sample of 516 Hindu and Muslim participants in rural West Bengal, India. Our treatments are the identity of the two players and the degree of religious fragmentation in the village where subjects reside. Our main finding is that the effect of social identity is small and inconsistent across the two religious groups in our study. While we find small but statistically significant results in line with our hypotheses in the Hindu sample, we find no statistically significant effects in the Muslim sample. This is in contrast to evidence from Chakravarty et al. (2016), who report significant differences in cooperation levels in prisoners’ dilemma and stag hunt games, both in terms of village composition and identity. We attribute this to the fact that social identity may have a more powerful effect on cooperation than on conflict.

## 1 Introduction

We often observe agents competing with each other to receive or get access to resources in a wide variety of economic and social situations. Examples of such contests include political competition, lobbying, or violent conflict. Resources spent in these contests are not often recoverable and have little social value. While competition between such groups can be resolved through the ballot box, often we also find such competition ending up in violence and civil wars [1, 2]. Given the loss of welfare, understanding the cause of such conflict can reduce the likelihood of conflicts.

Civil conflicts often occur between social and/or ethnic groups that compete for limited resources. A possible motivation for these social or ethnic groups to enter into socially expensive contests is that there are strong identities through which groups have ethnic preferences. These ethnic preferences can cause ethnic groups to restrict goods and services to members of their own ethnicity and deny them to other ethnic groups, thus resulting in conflicts. Social scientists have documented and analyzed such competition among social groups [3, 4].

A significant number of quantitative studies [5, 6] focus on aggregate cross-country analysis in order to explain violence. These cross-national studies find that the likelihood of wars and armed disputes among social groups increase with poverty and with weak institutions. More recently, there have been studies of competition and group violence using national-level data. Support for the increased competition for limited resources is found by Urdal [7] who shows that scarcity of productive resources and urban inequality increase the risk of armed conflict. Similarly, Mitra and Ray [4] also find that the improvement of economic status of a minority group can be perceived by the majority group as a threat, and can be a catalyst for conflict.

In this paper, we analyse to what extent social identity motivations can explain conflict at the individual level. It is well understood that social identity influences economic decisions [8, 9]. People’s preference for their own social group and or their bias against other social groups could lead greater competition and increased likelihood for conflict.

To this effect, we investigate what impact (if any) religious identities have on the likelihood of conflict over a resource using a lab-in-the-field experiment conducted in West Bengal, India. We study the effect of religious identity by comparing the behavior of Hindu and Muslim subjects when they play with their fellow in-group members to the case where they play with the out-group, i.e. someone belonging to a different religion. We furthermore study the effect of fragmentation on the likelihood of conflict by running experiments in villages where the overwhelming majority of the population is of one religion, as well as in villages where the population is roughly equally divided along religious lines.

Any individual likely identifies himself or herself with various identities: race, political affiliation, sexual orientation or religion shape our beliefs and actions [10, 11, 12]. Social groups formed from common links in race, religion and language can be more broadly classified as ethnic groups [13]. Here, we focus on one aspect of ethnicity: religion. In India, religion has a prominent position in society and it plays an important role in defining an individual’s identity. According to the Census of India 2001, Hinduism and Islam account for about 94% of India’s population (81% being Hindu and 13% Muslim). These religious groups have competed, often violently, in the past for resources, and continue to do so at present. This highlights the role religious identity could play in social and political spheres.

West Bengal, India, where we conduct our study, has witnessed several episodes of severe violence between these two religious groups. Bengal as a state has been partitioned twice along Hindu-Muslim lines: once by the British empire in 1905 and, on the occasion of independence, in 1947 when India and East Pakistan (now Bangladesh) were created. On both occasions there were mass displacements of people from one side of the newly created border to the other and widely documented inter-religious violence [14, 15]. Religious violence is still observed today, both in Bengal [16] and elsewhere [17]. The continuing violence and competition among the religious groups suggest that religious identity potentially plays a crucial role, especially in contexts where individuals perceive competition or threat for resources from members belonging to other religious groups. Some scholars argue that this competitive relationship between Muslims and Hindus stems from the historical power structure of the two groups. While most of the last millennium India’s political rulers belonged to the Muslim religion, up to 200 years prior to independence and since then, Muslims ceased to be the governing class [18].

In order to understand the effect of identity and social fragmentation on conflict and competition, we study the Tullock contest [19, 20]. In this game, each competing party can spend part of its wealth to increase the probability of obtaining a resource. However, expenditures are sunk and therefore non-recoverable to both winning and losing parties (see [21, 22] for reviews on the economics of conflict and contests, respectively).

There is a vast experimental literature on behavior in contests in experiments, recently reviewed by Dechenaux et al. [23]. The main finding from the literature on Tullock contest experiments is that subjects consistently bid above the risk neutral Nash equilibrium. In the overwhelming majority of the experiments done to date, individuals play the game in the absence of social context. While some experimental work has been done in the context of groups [24, 25, 26], these experiments study how individual effort provision changes when competition is done via groups. The fact that group effort is the sum of individual group members’ efforts introduces a public good problem, as there is the incentive to free ride on teammates.

Chowdhury et al. [27] study the role of identity in a three-player group Tullock contest in the lab. They consider artificial identities in the spirit of the minimal group paradigm, as well as real ethnic identities (South East Asian and Caucasian). The authors find that group expenditures in their control treatment are in excess of the risk neutral Nash equilibrium. However, unlike artificial identities, making ethnic identities salient leads to significant increases in effort.

Our paper contributes to this literature by considering the effect of group identity on behavior in single-player Tullock contests. We also study the effect of social fragmentation on behavior by postulating a saliency channel: religious identity should be more salient in fragmented villages and therefore, expenditure levels should be higher. Our main finding is that the effect of social identity is small and inconsistent across the two religious groups in our study. While we find statistically significant results in line with our hypotheses in the Hindu sample, the effect sizes are small. We find no statistically significant effects in the Muslim sample.

## 2 Experimental Design, Procedures and Hypotheses

### 2.1 The game

We implemented a simplified version of the Tullock contest. Subjects were endowed with INR 80, which they could spend to obtain a prize equal to INR 80. We set the prize value equal to the endowment to avoid the possibility of subjects incurring losses. The expected value of the contest, *V*_*i*_ is given by *V*_*i*_ = 80 + 80*p*_*i*_ − *E*_*i*_, *i* = 1, 2, where *E*_*i*_ ∈ {0, 20, 40, 60, 80} is the financial expenditure of player *i*. We opted for a reduced action set to facilitate participants’ understanding of the game. The probability of player *i* winning the contest is given by *p*_*i*_, which is equal to 1/2 if both players spend zero and *E*_*i*_/(*E*_*i*_ + *E*_*j*_) if at least one player spends a positive amount.

The payoff matrix in Table 1 displays the expected payoffs denominated in Indian Rupees (INR) in our experiment. The game was not displayed or explained to participants in this manner. See the following section on the experimental procedures, as well as the instructions and S1 Appendix for details. The unique Nash equilibrium of the game is (20, 20), in which both players bid a quarter of the value of the prize, as in the continuous version of the game. A somewhat unusual feature of our implementation of the Tullock contest is the assumption that if neither player makes a positive expenditure, both players have an equal chance of obtaining the prize. Note also that spending 40, 60 and 80 is strictly dominated by spending 20. Once we eliminate the strictly dominated strategies, we obtain a game with the same properties as the prisoners’ dilemma: the joint profit maximizing outcome is achieved when both players spend zero on the contest, but it is in their individual best interest not to do so. This does not have any implications on equilibrium behavior; behaviorally though, this could lead to a decline in average effort levels as compared to the extant literature.

**Table 1:** Expected payoffs for our implementation of the Tullock contest.

	0	20	40	60	80
0	40, 40	0, 60	0, 40	0, 20	0, 0
20	60, 0	20, 20	7, 13	0, 0	-4, -16
40	40, 0	13, 7	0, 0	-8, -12	-13, -27
60	20, 0	0, 0	-12, -8	-20, -20	-26, -34
80	0, 0	-16, -4	-27, -13	-34, -26	-40, -40

### 2.2 Experimental Design

The main purpose of the experiment is to understand how social identity preferences interact with religious fragmentation to affect behavior in Tullock contests. To understand the role of identity, we ran two treatments where subjects were playing with their fellow in-group members: one where Hindus were paired to play with other Hindus (H-H) with certainty, and another one where Muslims were paired with Muslims with certainty (M-M). We also ran a treatment where subjects were matched with an out-group: Hindus were paired with Muslims with certainty (H-M). Finally, we ran a treatment where there was a 50% chance a participant would be matched with someone of their own religion and a 50% he or she would be matched with someone of a different religion, which we denote as MIX. It was not possible for us to design a treatment in which identity was absent, since our experimental manipulation of religious identity relies on the names of all participants in the session being common knowledge, as well as the fact that subjects can observe the set of potential partners in the game. We explain in detail how we implemented these treatments and how we induced group identity in the subsection dedicated to experimental procedures below.

To understand the role of village-level fragmentation, we implemented M-M and H-H treatments in two types of villages: one in which one group accounted for at least 90% of the population, which we denote as homogeneous villages; and another type of village in which each religious group accounted for about 50% of the population of the village, which we denote as fragmented villages. Although the Indian Census collects village-level data on religious composition, that information is classified and not available to researchers. We use data from Das et al. [28] household survey in West Bengal on religious discrimination to select villages. Table 2 outlines the different treatments.

**Table 2:** Experimental design.

		Treatment			
		M-M	H-H	H-M	MIX
Village Type	Homogenous—Muslim	(94, 3)	-	-	-
	Fragmented	(40, 1)	(70, 2)	(130, 4)	(58, 2)
	Homogenous—Hindu	-	(124, 4)	-	-

Note: (# of subjects, # of villages).

### 2.3 Hypotheses

To develop our hypotheses, we will rely upon the simplified version of the model of other-regarding preferences proposed by [29], in which individuals exhibit disutility from obtaining payoffs either higher or lower than others. [29] also include a parameter *θ* to capture reciprocity concerns. Since reciprocity concerns do not play a role in our experiment, we exclude this parameter from the analysis. In a two-player setting, with players *i* and *j*, the utility function for player *i* takes the following form:

$$u_{i}\left(\pi_{j},\pi_{i}\right)=\left(\rho r+\sigma s\right)\pi_{j}+\left[1-\left(\rho r+\sigma s\right)\right]\pi_{i}.$$ (1)

The parameter *ρ* captures the extent to which player *i* cares about advantageous inequality, since *r* = 1 if *π*_*j*_ < *π*_*i*_ and 0 otherwise; it is referred to by [30] as capturing charity concerns. The parameter *σ* captures the extent to which player *i* cares about disadvantageous inequality, since *s* = 1 if *π*_*j*_ > *π*_*i*_ and 0 otherwise. [30] refer to this parameter as capturing envy. This formulation coincides with the model proposed by [31] if *σ* < 0 < *ρ* < 1 ([29], p. 823), but it can also encompass spiteful/competitive preferences if *σ* ≤ *ρ* ≤ 0.

Chen and Li [30] estimate the effect of group identity on other-regarding preferences using artificial identities in the spirit of the minimal group paradigm developed by [10]. Subjects were assigned to an artificial group and were asked to make a number of decisions. In each decision, subjects had to choose between two income distributions, whose recipients were (i) both in-group members, (ii) both out-group members, or (iii) one was an in-group member and the other was an out-group member. Chen and Li econometrically estimate *ρ* and *σ* conditional on the identity of the recipient. They find subjects in their experiment exhibit greater charity concerns and lesser envy towards in-group members than towards out-group members. In particular, their estimates are such that *ρ*_*I*_ > *ρ*_*O*_ > 0 and *σ*_*O*_ < *σ*_*I*_ < 0, where the subscript *I* indicates in-group and the subscript *O* denotes out-group.

We incorporate other-regarding preferences into the Tullock contest, this time using the more general specification proposed by [29]. The best response in the Tullock game by a player with Charness-Rabin preferences is given by:

$$BR_{i}\left(E_{j}\right)=\max\left\{-E_{j}+\frac{\left[1-2(\rho r+\sigma s)\right]}{1-(\rho r+\sigma s)}\sqrt{\frac{80E_{j}\left[1-(\rho r+\sigma s)\right]}{1-2(\rho r+\sigma s)}},0\right\}$$ (2)

The symmetric equilibrium of this game is given by

$$E_{i}^{*}=\frac{20-40(\rho r+\sigma s)}{1-(\rho r+\sigma s)}$$ (3)

If we assume that individuals are inequality averse (i.e. *σ* < 0 < *ρ* < 1), as has been found in the literature on other-regarding preferences to date, then similar to what [32] has shown for Fehr-Schmidt preferences, $\frac{\partial E_{i}^{*}}{\partial \sigma }>0$: an increase in “envy” concerns leads to higher effort. Conversely, lower “envy” leads to lower effort. Also, $\frac{\partial E_{i}^{*}}{\partial \rho }<0$: an increase in “charity” concerns leads to lower equilibrium effort and vice versa.

Having established our theoretical framework, we now apply it to our experimental design. We start by looking at the effect of identity preferences on behavior keeping religious composition fixed—focusing on fragmented villages. Our model predicts that there should be a relationship between the identity of the opponent in the Tullock contest and rent-seeking expenditures.

**Hypothesis 1:** *Expenditure levels in fragmented villages should increase in the probability of being matched with an out-group member.*

We now turn to the main hypothesis of the paper, which concerns the interaction between social identity and fragmentation. [33] proposes a theory of optimal distinctiveness, in which one’s affiliation to a group—and therefore our sense of identity—is affected by two competing needs. One one hand, we feel the need to belong to a group. On the other hand, we feel the need to be distinct. The former drives isolated individuals to seek membership of social groups, while the latter leads one to identify more strongly with groups that emphasize one’s uniqueness.

This theory therefore postulates that the degree of saliency of a particular identity will vary with how representative the members of the identity-relevant group are within a society. In his book on identity and conflict, [11] reiterates this point, when he argues that “[T]he importance of a particular identity will depend on the social context.” (p.25). Categories which provide a source of identity are naturally numerous, but Sen argues that meaningful identities are a small subset of the set of categories. They may become meaningful due to contextual specificity (i.e. national identity in the Olympics), or due to common circumstances which yield feelings of mutual solidarity (i.e. a natural disaster). Individuals consciously or unconsciously decide which identities they should assign greater weight when making decisions on a regular basis.

The corollary of this argument is that in settings where one religious group is predominant, individuals will put greater weight in other dimensions of their personal identity, since the religious domain of their identity does not provide sufficient distinctiveness, or is not sufficiently salient to provide the basis for meaningful trade-offs. In other words, our participants’ sense of religious identity should be more salient in villages where there is an out-group, as opposed to villages where all citizens share the same religious beliefs. A stronger sense of religious identity in fragmented villages therefore should imply that other-regarding preferences should be stronger in fragmented villages than homogeneous villages: $\sigma_{frag}^{I}<\sigma_{homog}^{I}$ and $\rho_{frag}^{I}>\rho_{homog}^{I}$. This in turn, leads to our final hypothesis.

**Hypothesis 2:** *Expenditure levels in H-H/M-M treatments should be lower in fragmented villages than in homogeneous villages.*

### 2.4 Participant Recruitment

We selected West Bengal to conduct our study for two reasons. Firstly, this Indian state has historically witnessed several episodes of inter-religious tension. The partition on Bengal along Hindu-Muslim lines in 1905 and the second partition of Bengal into West Bengal and East Pakistan (now Bangladesh) in 1947, when the modern Indian state was formed are particularly relevant to our study. In both cases, the mass displacements of people led to numerous episodes of inter-religious violence [14, 15]. There are numerous recorded incidents of violence between members of the two religious groups since the 1950s and the present day [4]. As recently as 2010, religious riots were recorded in West Bengal [16].

Secondly, our experimental design requires us to sample experimental participants from (and conduct our sessions in) two types of villages: villages where one religious group dominates, and villages whose population is roughly split along religious lines. Unfortunately, although the Indian Census does collect information on citizens’ religious affiliation, that data are not available to researchers at village level. Our sampling of the villages was instead based on data from [28], who conducted a large-scale household survey on religious fragmentation in West Bengal villages. Based on that survey, we labeled villages where 90% or more of the population was from one religious group as homogenous (they could be Homogenous-Muslim or Homogenous-Hindu), and villages were labeled as fragmented if they had no more than 60% of the village population from one group. Our choice of villages was further limited by the fact that we required a room that was big enough to hold 20–30 participants at a time for a few hours. The only such building in a village would be its primary school, which is where we conducted our experiments.

We employed a mixed-gender, mixed-religion team of local research assistants to recruit participants and conduct the sessions, so as to minimize any possible experimenter demand effect. A week ahead of a planned session, our research assistants travelled to the village where that session would take place. A set of neighborhoods were randomly selected, and within each neighborhood, recruitment was done on a door-by-door basis. On a given street, every two consecutive houses were skipped and the third house would be approached and those who agreed to participate would be signed up. Participants were reminded about the session the day before it took place. Participants did not know the purpose of the experiment: when approached, they were informed that the research team would be conducting decision-making sessions. We conducted one session per village. After the first session in the first village, it was clear that participants discussed the experiments among their social network. Due to a combination of the novelty factor and the generous incentive payments, the sessions themselves raised interest among villagers in the hours after the sessions ended, therefore contaminating the pool of potential participants in that village.

### 2.5 Experimental Procedures

We made religious identity salient by making the names of participants common knowledge, and by allowing participants to visually identify their potential counterparts in the games participants played. This is a combination of two existing methods of making identity salient: [34] induce ethnic identity in experiments conducted in Uganda using photographs of participants, while [35] induce ethnic identity in experiments conducted in Israel using participants’ names.

Upon arrival to the school building where the session was to take place, participants were asked to remain outside the main school building and wait for their name to be called out. Upon hearing their name, each participant was taken to the main classroom, and told to sit at one of the ends of the classroom, facing the middle. It is reasonably easy to identify someone as a function of their name, since Muslim names originate from Arabic, and are quite different from Hindu names. Calling in participants individually made their religious identities salient (and established the existence of an out-group) in an inconspicuous way. Eliciting religious identity through names could have also elicited participants’ caste identity as well. We control for this possibility in the econometric analysis of the data, and our results are robust.

Participants were told they would be making a series of decisions with someone on the other side of the room, and they were told that they would always make each decision with a different person. This allowed participants to identify the religious identity of their potential counterparts, either through their choice of attire, or by recognizing participants across the room. The experiments were unusual events in the villages, and many participants came to the sessions in formal attire. In rural Bengal, Hindu men wear “dhoti,” a long white cloth draped around the waist, and Muslim men wear “lungi,” a piece of checkered cloth also worn around the waist. Hindu women wear “saris,” as well as “bindi” on their forehead, while Muslim women wear “salwar” and “kamiz” and no “bindi”. However, since there were typically 15 to 20 participants on either side of the room, it was impossible for participants to know who their counterpart was in each game, therefore preserving the anonymity of decisions—this was important since 83% of participants stated in the post-experimental questionnaire that they recognized most of the participants in the room.

In the H-H and M-M sessions, all subjects in the room shared the same religion, so the seating arrangement was irrelevant. In the H-M sessions, Hindu subjects were all seated in one end of the room, while Muslim subjects sat in the other end; finally, in the MIX sessions, the experimenter team randomly allocated Hindu and Muslim subjects to either end of the room, subject to the constraint that an equal number of Hindu and Muslim subjects sat on either end of the room.

Sessions were split in three parts. In the first part, participants played three games: the Prisoners’ Dilemma, the Stag-Hunt game and the Tullock contest (in that specific order). In the second part of the session, participants played a series of individual decision-making tasks—the data from the Prisoners’ Dilemma and Stag Hunt game is the focus of a companion paper, [36]. In the third part, participants individually responded to a questionnaire in a separate room, got feedback on the decisions made in the experiment, and received their corresponding payment.

An experimenter standing in the middle of the room read the instructions aloud, using visual aids to explain the incentive structure of each game. We did not employ written instructions since about a third of our subjects was either unable to read or write, or could only write their name. As such, we denoted payoffs in INR and used images of Indian notes and coins to represent payoffs. This enabled these participants to fully understand the incentive structure of the game. See the S1 Appendix for copies of the instruction sets, the visual aids we used as part of explaining the game and decision forms.

The instructions explained the Tullock game as follows: subjects were told they would receive INR 80, which they could use to purchase lottery tickets. The lottery tickets would be put in a bag, along with the lottery tickets purchased by the other person they were matched with for that game. One ticket would be randomly drawn and the outcome would determine who would win the INR 80 prize. The actual draw was done at the end of the session for each pair. Each ticket cost INR 10, and subjects could purchase 0, 2, 4, 6 or 8 tickets. The framing of the experiment is consistent with the literature on Tullock contests and it was sufficiently familiar to subjects to allow them to understand the incentive structure.

A potential pitfall of running experiments in which subjects do multiple tasks is that there may be contamination of behavior across games, such as order effects, wealth effects, behavioral spillovers or hedging. Order effects are certainly possible in our experiment; while they might affect cooperation or rent-seeking levels, the hypotheses of interest are on differences in behavior across villages and/or treatments, all of which were exposed to the same order of play. We minimized the scope for wealth, spillover and hedging effects in our experiment by (a) not informing subjects of the games they were about to play ahead of time; (b) not providing feedback between games; (c) implementing a turnpike matching scheme, whereby subject *i* was never matched with the same person twice, and any of *i*’s matches would never play each other. Subjects were reminded of these features at the start of each game.

The first part of the session took approximately 60 minutes and sessions as a whole lasted on average 3 hours. The average payment for the whole session was INR 598.70 ($9.65). The average daily wage for a rural worker in West Bengal in 2011 ranged from INR 105 ($1.74) for an unskilled female worker to INR 297.50 ($4.93) for a male well digger; in most agricultural occupations average daily wages were approximately INR 130 ($2.15), Government of India (2012).

### 2.6 Ethics

Given that a substantial proportion of subjects could not read or write, we opted to administer a consent form verbally. Before the start of the session, an experimenter read a statement explaining that subjects’ decisions would be strictly anonymized, that all decisions would be identified only through an ID number, which would not be matched with their name.

Subjects were told they were free to leave the session at any time, and that they also had the right to opt out from the study and having their data removed from the study. An English language copy of the verbatim consent text is in S1 Appendix. This study was approved by the University of Exeter Ethics officer (IRB equivalent).

Consent was obtained by asking each subject to raise their hand if they objected to participating in the study. Since not all subjects could write, we could not record consent in written form; the experimenter team kept a register of subjects who declined to give their consent. Participants who were unwilling to proceed with the session, either after being read the consent statement, or at any point were free to leave and their data were removed from our database. This procedure was approved by the Ethics Officer overseeing this study.

We instructed our recruitment team not to recruit any participants under the age of 18. However, two participants reported in the post-experimental survey being 17 years old and another reported being 16 years old. We did not collect any identifying information from participants, including names, addresses, birth dates, or any identification numbers of any kind.

## 3 Results

**Fig 1:**
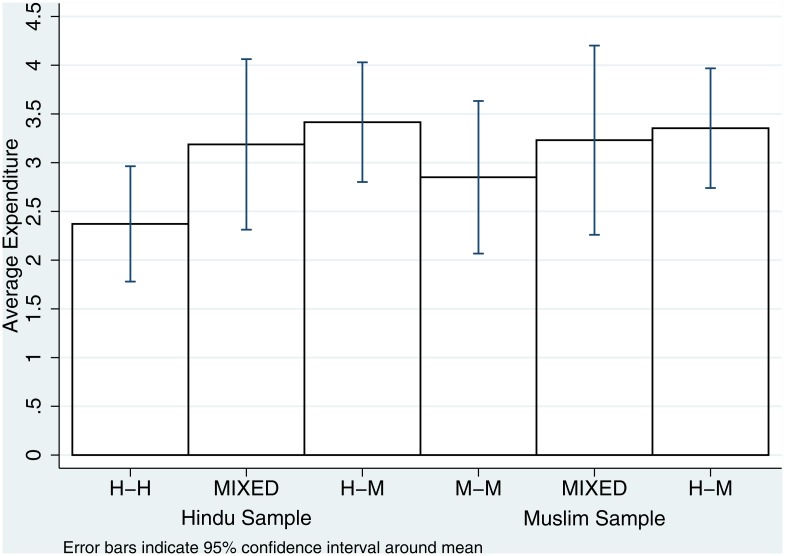
Average expenditure levels (measured by purchased tickets) in fragmented villages by treatment.

We start by testing Hypothesis 1. Fig 1 displays the average expenditure levels in each of the three conditions for the Hindu and Muslim samples, respectively. In both cases, average effort increases in the probability of being matched with an out-group member, although the differences are not large in absolute value. Furthermore, the 95% confidence intervals suggest some of these differences may not be statistically significant. Table 3 reports results from OLS estimations using the number of tickets purchased by participants as the dependent variable. Our results are robust if we use an ordered Logit estimator to account for the fact that our variable is ordinal and only takes five different values.

**Table 3:** OLS estimates of the determinants of expenditure in fragmented villages.

	Hindu Sample	Muslim Sample	Pooled Data	
DV: *E*_*i*_	(1)	(2)	(3)	(4)
Constant	2.37***	2.85***	2.37***	3.37***
	(0.30)	(0.39)	(0.30)	(1.26)
MIX	0.82	0.38	0.82	0.71
	(0.54)	(0.63)	(0.54)	(0.59)
H-M	1.04**	0.50	1.04**	0.96*
	(0.44)	(0.50)	(0.43)	(0.53)
M-M			0.48	0.74
			(0.50)	(0.65)
MIX × Muslim			0.04	0.04
			(0.66)	(0.75)
H-M × Muslim			-0.06	0.02
			(0.44)	(0.56)
Male				-0.50
				(0.43)
Married				-0.12
				(0.41)
Age				-0.01
				(0.01)
BornHere				-0.31
				(0.45)
PrimEdu				-0.31
				(0.47)
SecEdu				-0.68*
				(0.39)
TertEdu				-1.01
				(0.65)
DistHC				-0.03
				(0.04)
DisOG_*i*_				0.62
				(0.45)
DisOG_*i*_ × Muslim				0.21
				(0.69)
PropMyCaste				0.57
				(0.62)
KnowAll				0.16
				(0.41)
*R*^2^	0.04	0.01	0.03	0.06
*N*	167	131	298	298

Standard errors in parentheses. ***,**,*: *p* < 0.01, *p* < 0.05, *p* < 0.10.

We break up the results by sub-sample for ease of exposition, although we will consider the pooled data later on for subject pool comparisons. Regression (1) reports the estimation results of the restricted model on the Hindu sample. We find a positive and significant coefficient on H-M (*t* = 2.39, *p* = 0.018), though not on MIX (*t* = 1.51, *p* = 0.134); the coefficient on MIX is not significantly different to that of H-M (*F*(1, 164) = 0.17, *p* = 0.678). Regression (2) reports the estimation results of the restricted model on the Muslim sample. The results are quite different to those in the Muslim sample: no coefficient other than the constant (which relates to the omitted treatment, M-M) is statistically significant, which indicates there are no significant differences in average expenditure between M-M and MIX or H-M. We find no significant differences between H-M and MIX either (*F*(1, 128) = 0.05, *p* = 0.832).

Regression (3) considers the pooled Muslim and Hindu data, with the relevant interaction dummies, which confirm the analysis of regressions (1) and (2) with regards to treatment effects conditional on subject pool. Regression (4) adds controls at the individual level, such as age, gender, marital status and educational attainment; an attitudinal measure (DisOG_*i*_) measuring dislike of people of other religion, as well as a variable capturing the proportion of individuals of the same caste as the decision-maker among the pool of potential matches (i.e. those on the other side of the room), which we collected in the post-experimental survey. The sign and significance of the treatment coefficients on MIX and H-M are unchanged. The only coefficient on the control variables that is statistically significant is the coefficient on SecEdu (*t* = −1.71, *p* = 0.087); this is consistent with evidence from [37] on the negative correlation between subjects’ strategic sophistication (here proxied by educational attainment) and overbidding in the Tullock contest.

**Observation 1:** *In fragmented villages, Hindu participants spend a higher average amount in the Tullock contest when facing a Muslim participant than when facing a Hindu. We find no such difference among Muslims.*

**Fig 2:**
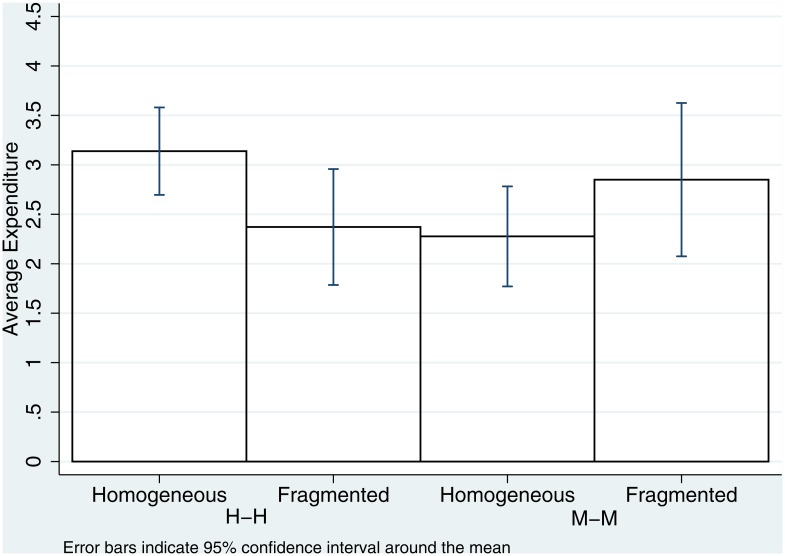
Average expenditure levels (measured by purchased tickets) in H-H and M-M treatment in fragmented and homogeneous villages.

We now turn to Hypothesis 2, which pertains to the effect of village composition on in-group biases. Fig 2 displays the average expenditures in the H-H and M-M conditions, varying the type of village where the sessions were conducted. We find, nominally at least, the opposite pattern across the two subject pools: among Hindus, average expenditure is lower in fragmented villages, while the opposite is true for Muslims. However, the absolute level of the difference is quite low: in both comparisons, the difference is around 0.5 tickets; recall that the purchasing unit in the experiment was two tickets.

Table 4 presents results from OLS estimates of the determinants of expenditure in H-H and M-M treatments in fragmented and homogeneous villages. We again break up the analysis by religious group, for ease of exposition. Column (1) presents the estimates of the reduced model for the Hindu sample. The constant coefficient, which corresponds to the average expenditure in homogeneous villages is positive and significant (*t* = 14.20, *p* < 0.001), while the Fragmented dummy coefficient is negative and significant (*t* = −2.09, *p* = 0.038). In other words, average expenditure by Hindus playing other Hindus in fragmented villages is significantly lower than in homogeneous villages. Column (2) presents the estimation results for the constrained model in the Muslim sample. The constant coefficient is positive and significant (*t* = 8.65, *p* < 0.001), but the coefficient on Fragmented is not significantly different from zero (*t* = 1.19, *p* = 0.236). In other words, there is no significant difference in average expenditure among Muslims playing against other Muslims across village types. Column (3) presents the combined model with a dummy variable for religious group and its interaction with M-M, which replicates the analysis of regressions 1 and 2 on the pooled data. Regression 4 presents the results from the unrestricted model with individual level controls. The coefficient on M-M is no longer significant (*t* = −1.22, *p* = 0.222); the only significant coefficient is that on TertEdu (*t* = −1.89, *p* = 0.059), again reiterating the earlier finding, this time at the tertiary education level.

**Table 4:** OLS estimates of the determinants of expenditure in in-group/in-group matches: fragmented vs. homogeneous villages.

	Hindu Sample	Muslim Sample	Pooled Data	
DV: *E*_*i*_	(1)	(2)	(3)	(4)
Constant	3.14***	2.28***	3.14***	3.53***
	(0.22)	(0.26)	(0.22)	(1.00)
Fragmented	-0.77**	0.57	-0.77**	-0.67*
	(0.37)	(0.48)	(0.37)	(0.40)
M-M			-0.86**	-0.58
			(0.34)	(0.48)
Fragmented × M-M			1.34**	1.24*
			(0.60)	(0.65)
Male				-0.47
				(0.36)
Married				-0.51
				(0.39)
Age				0.01
				(0.01)
BornHere				-0.44
				(0.39)
PrimEdu				0.08
				(0.46)
SecEdu				-0.37
				(0.38)
TertEdu				-1.07*
				(0.56)
DistHC				-0.01
				(0.03)
DisOG_*i*_				0.15
				(0.41)
DisOG_*i*_ × Muslim				-0.55
				(0.65)
PropMyCaste				0.17
				(0.55)
KnowAll				0.41
				(0.40)
*R*^2^	0.02	0.01	0.02	0.06
*N*	193	134	327	326

Standard errors in parentheses. ***,**,*: *p* < 0.01, *p* < 0.05, *p* < 0.10.

**Observation 2:** *Average expenditure by Hindu participants in the Tullock contest when playing against fellow in-group members are higher in homogeneous than in fragmented villages. We find no statistically significant difference in expenditure levels across village types among Muslims.*

We conclude by looking at differences in average expenditure across religious groups in fragmented villages. We would like to clarify that the model we are testing is *not* one of cultural or religious determinants of behavior in Tullock contests. As such, we do not have any *a priori* hypothesis to test. Nevertheless, there may be some value in exploring subject pool differences in average behavior conditional on a given treatment, particularly given that the only support for the theoretical predictions we do have comes from only one of the two subject pools. We start by looking at subject pool differences in the treatments conducted in fragmented villages. Column (3) in Table 3 presents a dummy interaction model, where a Muslim dummy (= 1 if participant *i* was Muslim) is interacted with the H-M and MIX dummies; we also add a dummy for the M-M treatment (which, in this case, is equivalent to having the Muslim dummy). The omitted category is therefore H-H. We do not find any subject pool differences in MIX (MIX × Muslim = 0: *t* = −0.52, *p* = 0.601) or in H-M (H-M × Muslim = 0: *t* = −0.81, *p* = 0.418). The coefficient on M-M is also non-significant (*t* = 0.96, *p* = 0.338), which indicates there was no difference in average expenditures between H-H and M-M. Adding individual controls in column (4) does not change the significance of the subject pool comparisons. Next, we look at subject pool differences in the homogeneous villages. Column (5) in Table 4 shows evidence of subject pool differences in homogeneous villages: the coefficient on M-M is negative and highly significant (*t* = −2.52, *p* = 0.012), indicating that Muslims in homogeneous villages playing in-group members spend less on the contest than Hindus in the same type of village. As a robustness check, we can also verify that the same is not true in fragmented villages: (Fragmented × Muslim = Muslim: *F*(1, 323) = 0.94, *p* = 0.333). Adding controls makes the difference between H-H and M-M in homogeneous villages no longer significant (*t* = −1.09, *p* = 0.277), while the difference between Muslim and Hindus in fragmented villages remains non-significant (*F*(1, 309) = 1.07, *p* = 0.302).

**Observation 3:** *We find significant level differences in average expenditures between Hindus and Muslims only when participants are playing with fellow in-group members in homogeneous villages.*

## 4 Discussion

The main finding of our experiment is that the average expenditure by subjects in our experiment appears to be sensitive to the identity of their match, or to the type of village in which they reside only in the case of the Hindu sample. We find relatively small, but statistically significant differences in average expenditure in the Hindu sample when we compare in-group/in-group matches to the treatment where it is certain that subjects will play an out-group; we also find significant differences between behavior in in-group/in-group matches across different village types. We find no significant differences in any treatment comparison in the Muslim sample.

We first want to place our results in context by comparing them to the extant literature on Tullock contests. We rule out the possibility that behavior in this experiment was somehow inconsistent with the typical behavior in this class of experiments. Average expenditure levels in our data are above the risk neutral Nash equilibrium, which is consistent with the literature (see [23] for an extensive review).

We now discuss the possible reasons why we find small treatment differences in our data. We start with methodological issues. Unlike the overwhelming majority of Tullock experiments, we considered a very coarse action set, in which participants could spend one of five different amounts, including zero. This design decision was made in order to make the game easier to explain to less well-educated participants. From a statistical point of view, the coarse action space could have inflated standard errors compared to the case where the same mean expenditure was drawn from a less coarse set of actions. Also, the coarse action set may have led to “bid compression”, in that for some subjects the optimal expenditure level might have been an intermediate, non-available level of expenditure (e.g. *E*_*i*_ = 3). Since that action was unavailable, subjects may have selected a lower level of expenditure. This in turn could have led to smaller effect sizes.

Another possible explanation for a possible bid compression and smaller effect sizes may have been the fact that participants played the Tullock game after having played the Prisoners’ Dilemma. [38] study behavioral spillovers between a linear public goods game and the Tullock contest. They find average expenditure in the Tullock contest is lower in the treatment where participants play in a parallel public goods game than the treatment where they play the Tullock contest in isolation. However, we still observed a large variation in effort levels across all treatments with 30–50% of all observations being in excess of the risk neutral Nash equilibrium (and strictly dominated strategies), so it is unlikely that bid compression is the primary reason for the absence of treatment effects.

A separate possibility is that participants’ strategic sophistication may have played an important role in determining behavior in the Tullock contest. [37] studies the extent to which individual characteristics determine bidding behavior in Tullock contests. He finds that individuals with lower cognitive ability are more prone to overbidding in the Tullock contest, although impulsivity is the main driver of behavior (pp. 19-20). We neither have measures of impulsivity or of cognitive ability; we do have a very crude proxy, which is educational attainment. We find a weakly significant correlation between educational attainment and overbidding, in that participants with secondary and/or tertiary education attainment bid less than illiterate participants. It is possible that, because the Hindu sample is more educated on average—both in our sample as well as in the West Bengal population—we were able to detect significant differences in the Hindu sample but not the Muslim sample. [37] also finds understanding of the experiment is negatively correlated with overbidding; we took care when designing the experimental protocol to ensure that participants understood the rules of the experiment. Further, the notion of a lottery would be quite familiar to participants.

In this light, it is interesting to contrast the behavior of our participants in the Tullock contest to their own behavior in the Prisoners’ Dilemma game, since when we eliminate strictly dominated strategies from the Tullock contest, both games share the same incentive structure (assuming risk neutrality). In our companion paper, [36], we documented significant differences in cooperation levels in the prisoners’ dilemma as a function of whether subjects play an in-group member or an out-group member, as well as whether subjects reside in a homogeneous or fragmented village. In particular, we found that in religiously-heterogeneous villages, cooperation rates in the Prisoners’ Dilemma were higher in in-group/in-group matches than in in-group/out-group matches. In addition, cooperation rates among in-group matches were significantly lower in homogeneous villages than in fragmented villages. Importantly, the results were largest in the case of the Muslim sample. We do not have a model of cultural differences to which we can resort to explain the sample differences in behavior across the cooperation and competition games we conducted. This is clearly an interesting open research question for the future.

[39] find that degree of social fragmentation has no effect on likelihood of civil war if per capita income and growth rates are controlled in the analysis, although controlling for village-level characteristics does not affect the sign of our average treatment effects. In this sense, it is possible that part of our results are driven by unobserved village characteristics, which we could not account for in our design—the test for our second hypothesis relies on a quasi-experimental design, in which village characteristics are taken as given. It is possible that different types of villages developed different norms over the course of generations, and this could prove to be confounded with our identity-saliency hypothesis. However, the same criticism applies to econometric estimation of observational data on conflict.

We conclude by comparing our evidence to other studies of identity on conflict. Hargreaves-Heap and Zizzo, (2009) show that out-group derogation can also be a powerful driver of behavior. [40] show that subjects make more competitive/spiteful choices when matched with out-group vs. in-group members, and there is evidence from the laboratory suggesting that competitive/spiteful preferences are correlated with higher expenditures in contests [37, 41]. [42] study the extent to which Kenyans exhibit coethnic bias in a series of lab-in-the-field experiments including measures of altruism (dictator game) and cooperation (public goods game). They find no evidence of coethnic bias. [27], who find a significant effect of real ethnic identities on behavior in three-player group Tullock contests. While our ability to draw parallels is limited by the fact that the strategic nature of the two games is slightly different (a group contest has a public good element which is absent in the single player case), there are still important insights to be gain from the comparison. [27] use East Asian students and Caucasian students in a UK university. As [11] argues, the saliency of one’s identity is a matter of context, and it is possible that the manipulation of identity in [27] was more effective in the laboratory setting. In their method, subjects were explicitly told that people in their group were of a particular ethnicity, and all others were of a different ethnicity. In a laboratory setting, most experimental cues are very salient, perhaps more than in the field. That, added to the fact that our religious manipulation was less direct may have resulted in group identities being more salient. This is an important methodological issue which merits further study.

## Supporting Information

S1 Appendix Table (A) Subject characteristics as a function of village type. (B) Ordered Logit estimates of the determinants of expenditure in fragmented villages. (C, D) Ordered Logit estimates of the determinants of expenditure in in-group/in-group matches: fragmented vs. homogeneous villages.
(PDF)
