# Supplementary material for: Religious Fragmentation, Social Identity and Conflict: Evidence from an Artefactual Field Experiment in India
Source: PLoS One. 2016 Oct 21;11(10):e0164708. doi: 10.1371/journal.pone.0164708 (PMC5074556; doi:10.1371/journal.pone.0164708)
Supplement: S1 Appendix — Table (A) Subject characteristics as a function of village type. (B) Ordered Logit estimates of the determinants of expenditure in fragmented villages. (C, D) Ordered Logit estimates of the determinants of expenditure in in-group/in-group matches: fragmented vs. homogeneous villages. (PDF) [file pone.0164708.s001.pdf]

# Appendix

## Subject Characteristics

In this section, we outline the basic characteristics of our sample. In particular, we wish to understand whether the participant subsample from homogeneous villages differs in a systematic way than the subsample from fragmented villages. To this effect, we compare the two types of villages, pooling the two types of homogeneous villages on a number of characteristics, including caste, marital status, place of birth (both the subjects and their next-of-kin), land ownership, profession, and literacy level.

Table A displays the proportion of subjects in each type of village that belong to each of 37 categories. We do not find large discrepancies on any category, although some of the differences are statistically significant using Fisher's exact test. The two differences that are worthy of note are the proportion of advantaged caste subjects, which is ten percentage points higher in Homogeneous villages, while OBC subjects are more prevalent by eight percentage points in Fragmented villages. We also sampled more subjects who either finished or were enrolled in tertiary education in Homogeneous villages. Nevertheless, we reiterate that we do not find systematic differences across multiple categories; even those categories where we there are significant differences, these are not sufficiently large to warrant concern.

## Methodological Note

Before reproducing the experimental materials, a methodological note is warranted. A large proportion of our participant sample was unable to read and/or write to a satisfactory level of proficiency. Around a third of our sample was completely unable to read or write and a further 17% only had basic primary education. As such, we had to describe the different games in a different way than that used in typical laboratory experiments. We took a number of design decisions, which we describe and justify in turn.

We opted not to present any payoff matrix to participants. Based on early pilots, we felt that a payoff matrix (even in simple $2 \times 2$ games) would be too confusing and abstract to many participants. Instead, we presented the game to participants using a simple, but familiar framing. We then enumerated the actions available to participants, and we described each contingency in the game in turn using visual aids. To circumvent the illiteracy problem, payoffs were described using rupee notes and coins, since all participants were familiar with currency.

We framed the Tullock contest as a task in which subjects were endowed with INR 80 and could spend any amount they wished on lottery tickets. Whatever lottery tickets were purchased would be put into a physical bag and one would be drawn. The winner would earn INR 80.

We piloted this frame in a session with a group of participants in the Birbhum district who had the same socio-economic background as our main subject pool. The feedback we obtained from post-session interviews suggested that our choice of framing led to participants understanding the incentive structure of the game without leading to experimenter demand effects. It is possible that our choice of framing could have led participants to interpret games in unintended ways, but we feel that participant confusion would be a worse outcome.

| Variable | Fragmented Villages |
|---|---|
| Male | 0.45 |
| Age | 35.24 (12.26) |
| SC | 0.18 |
| ST | 0.00 |
| OBC | 0.16 |
| Advantaged Castes | 0.60 |
| Single | 0.18 |
| Married | 0.77 |
| Widowed | 0.04 |
| Divorced | 0.01 |
| Separated | 0.00 |
| No Family Status | 0.00 |
| Born Here | 0.69 |
| Spouse Born Here | 0.42 |
| Father Born Here | 0.65 |
| Landless Contracted Labourer | 0.01 |
| Landless Farmer | 0.12 |
| Landless Non-contracted Labourer | 0.11 |
| Landed Less 0.5 H | 0.06 |
| Landed Less 1H | 0.08 |
| Landed More 1H | 0.03 |
| Seamstress | 0.09 |
| Student | 0.08 |
| Office Worker | 0.02 |
| Unemployed | 0.03 |
| Housewife | 0.26 |
| Attendant | 0.03 |
| Tutor House | 0.01 |
| Healthworker | 0.00 |
| Govt Rep | 0.03 |
| Quack | 0.01 |
| Tobacco Worker | 0.03 |
| Other | 0.01 |
| Retired | 0.00 |
| Illiterate | 0.20 |
| Sign Name | 0.13 |
| Primary Education | 0.15 |
| Secondary Education | 0.41 |
| Tertiary Education | 0.10 |

Standard deviations in paretheses.

p-values refer to 2-sided Fisher's exact tests except for "Age", where they refer to 2-sided t-test.

**Table A. Subject characteristics as a function of village type.**

## Instructions

The following instructions are the English translations from Bengali. Experimenters read them aloud to participants as a fixed script. The team of experimenters used large A1-sized sheets mounted in the middle of the room to assist them in explaining every contingency of each game. The text in bold inside square brackets indicates an action by the experimenter, and was not part of the script. We include the example sheets along with the main text for ease of exposition. We also include the decision forms in separate sub-sections.

### 0.0.1 Preamble

Welcome to our session. In this session, we will ask you to make series of decisions. This session is part of a large study sponsored by a university. The purpose of this study is to understand how people make decisions in a typical Indian village. The objective is to better understand how to improve the welfare of villagers in India.
The decisions you will make are not a test of your knowledge. There is no right or wrong way to decide. What we want to know is how you decide when faced with slightly different problems. These problems give you the chance of earning a significant amount of money, so please think carefully before making your decisions.
Please do not talk either to the people sitting next to you or the people across the room about the task. If you have any questions about the experiment, or if something does not make sense, please raise your hand, and one of my colleagues will take your question.
The money you earn will depend on what you choose, on what other people in the room choose and sometimes depending on chance.
We will first explain to you carefully the nature of each decision, and how your payment is determined in each decision. This will involve some examples. Please pay attention to the rules. If you have any question or if the rules are difficult to understand, please ask. It is very important to us that you understand how each decision works.
You will make your decisions on a piece of paper, which we will provide. Please make sure you fill all the necessary decisions, since these will be what determines your payment for the session.
The pieces of paper you will receive will have a number. This number is unique to you. We will pay you based on your number. Please do not write your name on the piece of paper. That way, no one will ever be able to link the decisions you make in this session to you.
Your payment for each task will be determined at the end of the session. You will then be paid in cash. While you are collecting your cash we will also do a brief questionnaire with each of you individually.

### 0.0.2 Tullock Contest Instructions

In this task you will be paired with someone across the room. You will only be paired with that person for this game; you will never be paired with that person again in this session. You and person with whom you are matched will have to make a decision. Your payment for this task will depend on what you choose and what the other person chooses. We will give you 80 rupees. You may use any of the 80 rupees to bid for a prize. This prize is worth 80 rupees. The prize will be drawn in a lottery. To win, you must buy tickets; each ticket costs 10 rupees. You can buy 0, 2, 4, 6 or 8 tickets. We will place each ticket you buy in a bag; each ticket the other person buys, we will place it in the same bag. We will draw one ticket at random; if that ticket is yours, you

receive the prize; if not, the other person receives the prize. Note our lottery is slightly different from regular lotteries you might be familiar with. Unlike regular lotteries, in our lottery the total number of lottery tickets is NOT fixed. Therefore, while like regular lotteries, in our lottery too the more tickets you buy, the higher the chance you have to win. However, unlike regular lotteries, in our lottery, the more tickets the other person buys, the higher the chance you will lose. Remember, once you buy the tickets you cannot have the money you spent on them back, whether you win the prize or not. If you win, your payment for this task will be 80 rupees minus what you spent plus the prize. If you lose, your payment for this task will be 80 rupees minus what you spent. Both you and the other person must choose at the same time. This means you will not know what the other person has chosen while making your own choice. Let?s go through a few examples using my colleagues. **Example 1:**

The sheet on the wall shows the first example we would like to go through with you. Suppose [X] buys 6 tickets and [Y] buys 4 tickets. This means there 10 tickets in the bag. [X] has a 6-in-10 chance of winning the prize and [Y] has a 4-in-10 chance of winning. If the ticket that is drawn is [X]?s, he will win the prize. [X]?s final payment is, [**TRY TO ELICIT ANSWER FROM A PARTICIPANT!**] the value of the prize (80 rupees), plus the 20 rupees he kept = 100 rupees. [Y]?s final payment is, [**TRY TO ELICIT ANSWER FROM A PARTICIPANT!**] the 40 rupees she kept. If the ticket that is drawn is [Y]?s, she will win the prize. In that case [X]?s final payment is, [**TRY TO ELICIT ANSWER FROM A PARTICIPANT!**] the 20 rupees he kept. [Y]?s final payment is [**TRY TO ELICIT ANSWER FROM A PARTICIPANT!**] 80 rupees plus the 40 rupees she kept = 120 rupees. **Example 2:**

The sheet on the wall shows the second example we would like to go through with you. Suppose [X] buys 6 tickets and [Y] buys 8 tickets. This means there are 14 tickets in the bag. [X] has a 6-in-14 chance of winning the 80 rupees and [Y] has a 8-in-14 chance of winning. If the ticket that is drawn is [X]?s, he will win the prize. [X]?s final payment is, [**TRY TO ELICIT ANSWER FROM A PARTICIPANT!**] the value of the prize 80 rupees, plus the 20 rupees he kept = 100 rupees. [Y]?s final payment is, [**TRY TO ELICIT ANSWER FROM A PARTICIPANT!**] 0 rupees since she did not keep any rupees from the original amount she had. If the ticket that is drawn is [Y]?s, she will win the 80 rupees. [X]?s final payment is, [**TRY TO ELICIT ANSWER FROM A PARTICIPANT!**] the 20 rupees he kept. [Y]?s final payment is, [**TRY TO ELICIT ANSWER FROM A PARTICIPANT!**] 80 rupees. **Example 3:**

The sheet on the wall shows the third example we would like to go through with you. Suppose [X] buys 8 tickets and [Y] buys 8 tickets. This means [X] will have 8 tickets in the bag and [Y] will have 8 tickets in the bag. This means [X] has a 1-in-2 chance of winning the prize and [Y] also has a 1-in-2 chance of winning. If the ticket that is drawn is [X]'s, he will win the prize. [X]'s final payment is, [**TRY TO ELICIT ANSWER FROM A PARTICIPANT!**] the value of the prize, 80 rupees, since he did not keep any rupees from the original amount he had. [Y]?s final payment is, [**TRY TO ELICIT ANSWER FROM A PARTICIPANT!**] 0 rupees, since she did not keep any rupees from the original amount she had. If the ticket that is drawn is [Y]?s, he will win the prize. [X]'s final payment is, [**TRY TO ELICIT ANSWER FROM A PARTICIPANT!**] 0 rupees, since he did not keep any rupees from the original amount he had. [Y]?s final payment is, [**TRY TO ELICIT ANSWER FROM A**

**PARTICIPANT!**] 100 rupees. **Example 4:**

The sheet on the wall shows the fourth example we would like to go through with you. Suppose [X] buys 0 tickets and [Y] buys 0 tickets. Since no person bought any ticket, we flip a coin to determine who wins the prize. This means [X] has a 1-in-2 chance of winning the prize and [Y] also has a 1-in-2 chance of winning.
If [X] wins, [**TRY TO ELICIT ANSWER FROM A PARTICIPANT!**] he will receive the prize plus the 80 rupees he started with which adds to 160 rupees. [Y] will receive, [**TRY TO ELICIT ANSWER FROM A PARTICIPANT!**] the 80 rupees she started with. If [Y] wins he will receive, [**TRY TO ELICIT ANSWER FROM A PARTICIPANT!**] the prize plus the 80 rupees he started with which adds to 160 rupees. [X] will receive, [**TRY TO ELICIT ANSWER FROM A PARTICIPANT!**] the 80 rupees she started with. **Example 5:**

The sheet on the wall shows the fifth example we would like to go through with you. Suppose [X] buys 2 tickets and [Y] buys 0 tickets. This means [X] will have 2 tickets in the bag, while [Y] will have no tickets in the bag. Hence, [X] will win and his payment will be, [**TRY TO ELICIT ANSWER FROM A PARTICIPANT!**] the prize plus the 60 rupees he has left. His total payment is 140 rupees. [Y] will receive , [**TRY TO ELICIT ANSWER FROM A PARTICIPANT!**] 80 rupees. ANY QUESTIONS? (wait for a few seconds) In your decision sheet, please choose how many lottery tickets you want to buy, where each lottery ticket costs you 10 rupees. [Experimenters should now hand the decision sheet to the subjects]

## Post-experimental Questionnaire

After all participants completed the final task and the experimenter team collected all decision materials, participants were called individually to a separate room where they were asked a number of survey questions, prior to knowing the outcome of each game and receiving their payoff. Table B outlines each question, along with summary statistics.

| Question Text | Category/Domain |
|---|---|
| Age | $[16, 80]$ |
| Religion | {Hindu, Muslim, Christian, Sikh, Buddhist, Parsi, Other} |
| Caste | {SC, ST, OBC, Normal, Other} |
| Marital Status | {Single, Married, Widowed, Divorced, Husband Left, Other} |
| Born in Village? | {Yes, No, Don't Know} |
| If not, how long have you lived here? | |
| Spouse Born in Village? | {Yes, No, Don't Know} |
| If not, how long has (s)he lived here? | |
| Father Born in Village? | {Yes, No, Don't Know} |
| If not, how long has he lived here? | |
| Grandfather Born in Village? | {Yes, No, Don't Know} |
| If not, how long has he lived here? | |
| Education Level | {Illiterate, Sign Name, Dropped out at grade $x$, Completed grade $x$, Currently sitting grade $x$ } |
| Profession | {Landless contract laborer, Landless farmer, Landless non-contract laborer, Attendant, Small-property farmer ($<$ 0.5 Ha), Medium-property farmer ($<$ 1 Ha), Big-property farmer $>$ 1 Ha), Quarry worker, Student, Office worker, Unemployed, Housewife, Tutor House, Health Worker, Gov't employment program, Village quack, Village tobacco factory, Other} |
| Does your village have a pond? | {Yes, No, Don't Know} |
| Who owns it? | {Gov't, NGO, Village} |
| Do you use it? | {Yes, No, Don't Know} |
| Has it been appropriated/expropriated? | {Yes, No, Don't Know} |
| If yes, by whom? | {Higher caste, Land-owning villagers, Rich families, Political party, Panchayat, Other} |
| Does your village have a tubewell? | {Yes, No, Don't Know} |
| Who owns it? | {Gov't, NGO, Village, Private individual, Don't Know} |
| Do you use it? | {Yes, No} |
| Has it been appropriated/expropriated? | {Yes, No, Don't Know} |
| If yes, by whom? | {Higher caste, Land-owning villagers, Rich families, Political party, Panchayat, Other} |

| | |
|---|---|
| How far is the Block Health Center? | |
| If you fall ill, where do you go? | {Dispensary, Primary Health Center,<br>Block Health Center, District Hospital,<br>Nursing Home, Private Doctor,<br>Village Quack, Other} |
| Name 3 public goods your village lacked<br>for the last 3 years | {Water, Education, Health, Transport,<br>Road, Drainage, No Problems,<br>Don't Know, Others } |
| Name 3 important public goods | {Water, Education, Health, Transport,<br>Road, Drainage, No Problems,<br>Don't Know, Others} |
| Do you think of yourself as an Indian? | {Yes, No, Indifferent, Don't know,<br>I belong to this village/district} |
| Do you think of yourself as a Hindu/Muslim? | {Yes, No, Indifferent, Don't know} |
| Do you believe you belong to this village? | {Yes, No, Indifferent, Don't know} |
| If a close relative married a non-hindu/<br>non-muslim, how would you feel? | {Good, Bad,<br>Indifferent, Not Bad, Don't know} |
| If your neighbor belongs to a different religion,<br>how would you feel? | {I like, I don't like, It's normal,<br>Do not dislike, Indifferent,<br>We do not mix, Don't know} |
| (Hindus only) If your neighbor belongs to a<br>different caste, how would you feel? | {I like, I don't like, It's normal,<br>Do not dislike, Indifferent,<br>We do not mix, Don't know} |
| Would you like children from other religions<br>in your child's school? | {Few, < half, Half,<br>> Half, Almost everyone,<br>I don't like children from other<br>religions in school,<br>Better everyone studies together,<br>Don't know} |
| In your village, how many are of your religion? | {Few, < Half, Half, > Half,<br>Almost everyone, Don't know} |
| In today's session, was there any person from<br>your religion or other religion whom you<br>personally knew? | {Few, < Half, Half, > Half,<br>Almost everyone, Don't know} |

**Table B. Post-experimental Questions.**

# 1 Ordered Logit counterparts to OLS regressions

| | Hindu Sample | Muslim Sample | Pooled Data | |
|---|---|---|---|---|
| DV: $E_i$ | (1) | (2) | (3) | (4) |
| Cutoff 1 | -0.92 | -1.03 | -0.86 | -1.46 |
| | (0.23) | (0.31) | (0.22) | (0.91) |
| Cutoff 2 | 0.87 | 0.39 | 0.77 | 0.20 |
| | (0.23) | (0.30) | (0.22) | (0.91) |
| Cutoff 3 | 1.56 | 1.52 | 1.66 | 1.11 |
| | (0.25) | (0.33) | (0.24) | (0.91) |
| Cutoff 4 | 2.31 | 2.51 | 2.52 | 1.99 |
| | (0.30) | (0.39) | (0.27) | (0.92) |
| MIX | 0.48 | 0.34 | 0.51 | 0.52 |
| | (0.39) | (0.45) | (0.39) | (0.42) |
| H-M | $0.56^{*}$ | 0.44 | $0.57^{*}$ | 0.62 |
| | (0.32) | (0.36) | (0.31) | (0.40) |
| MIX × Muslim | | | 0.08 | 0.43 |
| | | | (0.47) | (0.46) |
| H-M × Muslim | | | 0.10 | 0.06 |
| | | | (0.32) | (0.53) |
| M-M | | | 0.25 | 0.14 |
| | | | (0.35) | (0.41) |
| Male | | | | -0.31 |
| | | | | (0.31) |
| Married | | | | -0.14 |
| | | | | (0.29) |
| Age | | | | -0.01 |
| | | | | (0.01) |
| BornHere | | | | -0.30 |
| | | | | (0.32) |
| PrimEdu | | | | -0.26 |
| | | | | (0.34) |
| SecEdu | | | | -0.48 |
| | | | | (0.29) |
| TertEdu | | | | -0.54 |
| | | | | (0.46) |
| DistHC | | | | -0.02 |
| | | | | (0.03) |
| $\text{DisOG}_i$ | | | | 0.38 |
| | | | | (0.32) |
| $\text{DisOG}_i$ × Muslim | | | | 0.18 |
| | | | | (0.49) |
| PropMyCaste | | | | 0.52 |
| | | | | (0.46) |
| KnowAll | | | | 0.15 |
| | | | | (0.29) |
| *LL* | -242.93 | -199.92 | | -440.70 |
| *N* | 167 | 131 | 298 | 298 |

Standard errors in parentheses. $^{***}, ^{**}, ^{*}: p < 0.01, p < 0.05, p < 0.10$.

**Table C. Ordered Logit estimates of the determinants of expenditrure in fragmented villages.**

| | Hindu Sample | Muslim Sample | Pooled Data | |
|---|---|---|---|---|
| DV: $E_i$ | (1) | (2) | (3) | (4) |
| Cutoff 1 | -1.27 | -0.44 | -1.20 | -1.53 |
| | (0.20) | (0.21) | (0.19) | (0.75) |
| Cutoff 2 | 0.30 | 0.95 | 0.29 | -0.0003 |
| | (0.18) | (0.22) | (0.17) | (0.74) |
| Cutoff 3 | 1.12 | 1.74 | 1.11 | 0.83 |
| | (0.20) | (0.26) | (0.18) | (0.75) |
| Cutoff 4 | 2.26 | 2.40 | 2.05 | 1.79 |
| | (0.27) | (0.32) | (0.23) | (0.76) |
| Fragmented | -0.44$^{*}$ | 0.50 | -0.42 | -0.39 |
| | (0.27) | (0.34) | (0.26) | (0.28) |
| M-M | | | -0.70$^{***}$ | -0.66$^{*}$ |
| | | | (0.26) | (0.35) |
| M-M $\times$ Fragmented | | | 0.94$^{**}$ | 1.06$^{**}$ |
| | | | (0.43) | (0.46) |
| Male | | | | -0.27 |
| | | | | (0.26) |
| Married | | | | -0.38 |
| | | | | (0.29) |
| Age | | | | 0.01 |
| | | | | (0.01) |
| BornHere | | | | -0.30 |
| | | | | (0.28) |
| PrimEdu | | | | 0.02 |
| | | | | (0.34) |
| SecEdu | | | | -0.29 |
| | | | | (0.29) |
| TertEdu | | | | -0.71$^{*}$ |
| | | | | (0.42) |
| DistHC | | | | -0.02 |
| | | | | (0.02) |
| DisOG$_i$ | | | | 0.04 |
| | | | | (0.29) |
| DisOG$_i$ $\times$ Muslim | | | | -0.25 |
| | | | | (0.47) |
| PropMyCaste | | | | 0.52 |
| | | | | (0.43) |
| KnowAll | | | | 0.26 |
| | | | | (0.28) |
| *LL* | -286.24 | -190.60 | -478.40 | -472.03 |
| *N* | 193 | 134 | 327 | 326 |

Standard errors in parentheses. $^{***}, ^{**}, ^{*}: p < 0.01, p < 0.05, p < 0.10$.

**Table D. Ordered Logit estimates of the determinants of expenditure in in-group/in-group matches: fragmented vs. homogeneous villages.**
